# Xuesaitong Soft Capsule (Chinese Patent Medicine) for the Treatment of Unstable Angina Pectoris: A Meta-Analysis and Systematic Review

**DOI:** 10.1155/2013/948319

**Published:** 2013-12-30

**Authors:** Xiaochen Yang, Xingjiang Xiong, Heran Wang, Guoyan Yang, Jie Wang

**Affiliations:** ^1^Department of Cardiology, Guang'anmen Hospital, China Academy of Chinese Medical Sciences, Beixiange 5, Xicheng District, Beijing 100053, China; ^2^Cancer Research Institute, Central South University, Changsha, Hunan 410078, China; ^3^Centre for Evidence-Based Chinese Medicine, Beijing University of Chinese Medicine, Beijing 100029, China

## Abstract

*Objective.* To provide a systematic review to evaluate the effectiveness and safety of Xuesaitong soft capsule (XST) in treating unstable angina (UA). *Methods. *An extensive search of 6 medical databases was performed up to August 2013. Randomized controlled trials (RCTs) involving XST alone or combined with conventional drugs versus conventional drugs were included. A meta-analysis of reduction of angina symptoms and electrocardiogram (ECG) improvement was performed to evaluate the effects of XST on UA. *Results.* After researching, a total of 6 RCTs with 716 participants were included. Our review showed that XST combined with conventional drugs had significant effect on relieving angina symptoms (RR: 1.14 [1.07, 1.22]; *P* = 0.0001) and improving ECG (RR: 1.26 [1.12, 1.42]; *P* = 0.0001) compared with conventional drugs alone. *Conclusions.* XST appears to have beneficial effects on improvement of ECG, reduction of angina symptoms, and decreasing the frequency and duration of angina attack in participants with UA. However, the findings should be interpreted with caution due to the poor methodological quality of the included trials.

## 1. Introduction

Coronary artery disease (CHD) is one of the leading causes of death in most developed and some developing countries [1, 2]. Antiplatelet agents and anticoagulants have demonstrated variable clinical effects in coronary heart disease (CHD), including unstable angina pectoris (UA) and acute myocardial infarction (AMI) [3, 4]. Among the antiplatelet agents, aspirin has been shown to reduce the risk for thrombosis and ischemic events. However, the possibility of aspirin resistance, which has been described as a number of phenomena, including antithrombotic complications, prolongation of the bleeding time, and inhibition of thromboxane biosynthesis, provides an impetus for researching new medicine with high effectiveness and fewer adverse effects [5].

Sanqi, also known as radix notoginseng, is a hemostatic herbal medicine that may have protective effect in patients with UA [6] and has been used for cardiovascular diseases for hundreds of years in China. Recently, with the growing and sustained interest in the benefits of Chinese herbal medicine (CHM) and potential drug interactions with western medications, Sanqi called much attention for its good cardiovascular effects, including inhibition of platelet aggregation, increasing blood flow, improving left ventricular diastolic function in hypertensive patients, and anti-inflammatory effect [7, 8]. *Radix notoginseng* consists of complex compounds, including saponin, dencichine, polysaccharides, amino acids, flavonoids, phytosterols, fatty acids, volatile oils, aliphatic acetylene hydrocarbons, and trace elements. *Panax notoginseng saponins* (PNS) are one of the main active ingredients of Radix notoginseng. Until now, twenty-seven saponins were identified and nine of them were notoginsenoside R1, ginsenoside Rb1, Rb2, Rb3, Rc, Rd, Re, Rf, and Rg1 [9, 10]. Most of these monomer components are 20(S)-protopanaxadiol and 20(S)-protopanaxatriol. Xuesaitong soft capsule (Kunming Samflaming Pharmacy Group Co., Ltd., Kunming, China) mainly consists of PNS. The total dose is 100–200 mg for one day. Modern researches have shown that Xuesaitong soft capsule (XST) can stanch bleeding, invigorating and supplementing blood for treating UA [11, 12].

Several lines of evidence published in China have reported that XST can enhance the effect of relieving of angina symptoms, decreasing the dosage of nitroglycerin, and lessening adverse effects for patients with UA [19–22]. However, the lines of evidence supporting or disproving these cardiovascular effects have not been well systematically reviewed. In this paper, we evaluated the effect of XST through a rigorous systematic review and meta-analysis of randomized trials.

## 2. Methods 

### 2.1. Study Selection and Search Strategy

The following electronic databases including PubMed, the Cochrane Center Controlled Trials Register (2013), EMBASE (1980–2013), Chinese National Knowledge Infrastructure (CNKI, 1979–August 2013), Chinese Biomedical Literature Database (CBM, 1978–August 2013), Chinese Scientific Journal Database (VIP, 1989–August 2013) were searched by two authors (X. Yang and H. Wang). The titles and abstracts of potentially relevant studies were identified through searching and reviewed independently by 3 reviewers (G. Yang, H. Wang, and X. Xiong). We selected randomized controlled trials that evaluate the cardiovascular effects of XST for UA. The English searching terms were used individually or combined including “unstable angina pectoris,” “Xuesaitong soft capsule,” “randomized controlled trial,” “controlled clinical trial,” “randomly,” “trial,” “randomised” and “randomized.” The Chinese searching terms were used individually or combined including those for the generic name of unstable angina pectoris (“Bu_wen_ding_xing_xian_jiao_tong”), Xuesaitong soft capsule (“Xue_sai_tong_ruan_jiao_nang”), and randomized (“sui_ji”). For discrepancies in the process of selection, whether to include or exclude a study was resolved by consensus with other investigator (X. Yang).

### 2.2. Types of Interventions and Participants

The included trials were designed to compare the effectiveness and safety of XST used alone or in combination with conventional drugs versus conventional drugs alone. The intervention group was treated with XST alone or combined with conventional drugs for patients with UA regardless of manufactures, dose, and duration. The participants who are suffering from and have been diagnosed with UA should be included regardless of the severity. The diagnosis of UA [23, 24] was according to “ACCF/AHA Guideline for the Diagnosis and Management of Patients with Unstable Ischemic Heart Disease (ACCF/AHA)” or “the International Society and Federation of Cardiology/World Health Organization (ISFC/WHO)”.

### 2.3. Types of Outcome Measures

According to the severity and treatment of diseases, the primary outcome measures were mortality due to ischemic heart disease or incidence of heart events (e.g., AMI, severe arrhythmia, heart failure, and revascularization). The secondary outcome measures were: (1) reduction of angina symptoms (e.g., effective symptomatic improvements should achieve at least 50% or 80% reduction in frequency of feeling of angina chest pain [24, 25]), (2) Electrocardiogram (ECG) improvement (e.g., effective ECG improvements should achieve at least 0.05 mV lowering at ST segment in ECG or nearly normal ECG during an exercise test [24, 25]), (3) frequency of angina attack, and (4) duration of angina attack. The followup and adverse events were also measured.

### 2.4. Data Extraction and Quality Assessment

The qualities of included RCTs were assessed according to several specific domains including random sequence generation, allocation concealment, blinding of participants and personnel, blinding of outcome assessment, incomplete outcome data, and selective reporting. Two authors (H. Wang, G. Yang) independently assessed the methodological quality of included RCTs according to Cochrane Handbook: such information was provided: (a) a description of included study and (b) their judgment on the risk of bias as a consequence of this. This judgment is categorized by using one of the three answers: “high risk,” “unclear risk,” and “low risk”. About the included RCTs, the following data were extracted: authors of study, year of publication, sample size and age of participants, detailed information of interventions and controls, outcome measures, and adverse events.

### 2.5. Statistical Analysis

Meta-analyses of RCTs were performed by using RevMan 5.1 software. We summarized data using risk ratios (RR) with 95% confidence intervals (CI) for dichotomous outcomes or mean difference (MD) with 95% CI for continuous outcomes. We assessed heterogeneity using both the Chi-squared test and the *I*-squared statistic. If *I*-squared value was greater than 25%, we pooled data using a fixed-effect model; otherwise we use random effects model [26].

## 3. Result

### 3.1. Description of Studies

**Figure 1:**
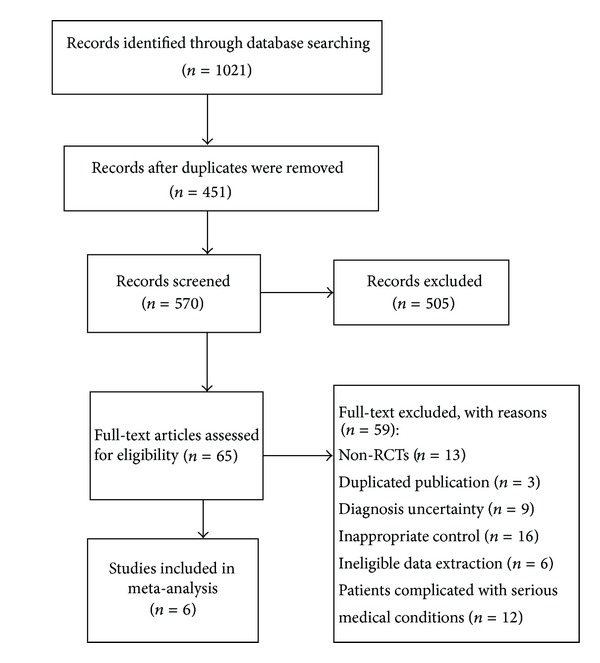
Flow chart of study search and selection.

A flow chart (Figure 1) showed the search process and study selection. We included 6 RCTs [13–18] for this systematic review. All RCTs were conducted in China and published in full. The detailed characteristics of the included trials were listed in Table 1. The 6 RCTs involved 716 participants with UA, aged between 37 and 77. The duration of treatment varied from 4 weeks to 8 weeks, with an average duration of 4.6 weeks. The dosage of XST was two capsules twice a day. There was one comparison: XST plus conventional drugs versus conventional drugs. Reductions in angina symptoms and improvement in ECG were the most commonly measured outcomes in the included studies. One trial [15] adopted mortality as primary outcome.

### 3.2. Risk of Bias Assessment

The included studies indicated randomization with a single-center, parallel design, but most of them did not describe it clearly. Only one trial [13] reported that the random sequence was generated by random number table. None of the trials describe allocation concealment and methods of assessing compliance. One trial [15] reported dropout or withdrawal, but none of the trials had a pretrial estimation of sample size, which indicated the lack of statistical power to ensure appropriate estimation of the therapeutic effect.

### 3.3. Effects of Interventions

#### 3.3.1. Primary Outcomes

After four months of followup, one trial [15] reported 1 case of severe arrhythmia in intervention group and 2 cases of AMI in control group.

#### 3.3.2. Secondary Outcomes 

**Figure 2:**
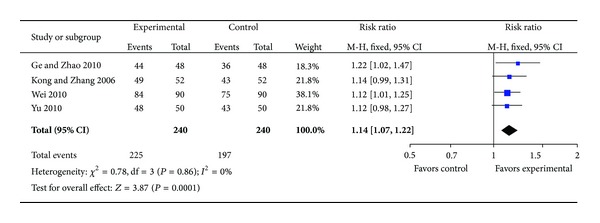
Forest plot of comparison: XST plus conventional drugs versus conventional drugs, outcome: RAS.

*(i) Reduction of Angina Symptoms (RAS).* Compared with conventional medicine, four individual trials [15–18] with 218 patients reporting reduction of angina symptoms after treatment favored XST plus conventional medicine. Homogeneity in the results is showed (*P* = 0.86, *I* ^2^ = 0%). Thus, we did a quantitative data synthesis (meta-analysis) by fixed-effects model. The outcome shows a statistically significant difference in favor of the combination group of XST and conventional drugs (RR: 1.14 [1.07, 1.22]; *P* = 0.0001). It is suggested that XST plus conventional drugs had a better effect on relieving symptoms of angina (Figure 2).

**Figure 3:**
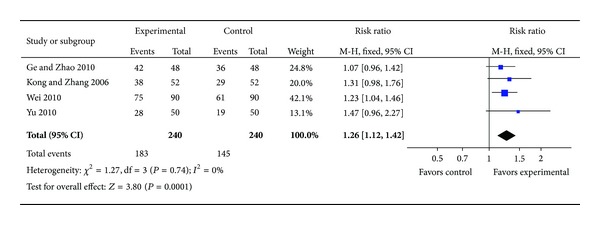
Forest plot of comparison: XST plus conventional drugs versus conventional drugs, outcome: ECG.

*(ii) ECG Improvement.* Compared with conventional medicine, four individual trials [15–18] with 218 patients reporting ECG improvement after treatment favored XST plus conventional medicine. Homogeneity in the results is showed (*P* = 0.74,  *I* ^2^ = 0%). Thus, we did a quantitative data synthesis (meta-analysis) by fixed-effects model. The outcome shows a statistically significant difference in favor of the combination group of XST and conventional drugs (RR: 1.26 [1.12, 1.42]; *P* = 0.0001). It is suggested that XST plus conventional drugs had a better effect on improving ECG (Figure 3).

*(iii) Frequency of Angina Attack.* Compared with conventional medicine, four individual trials [13, 14, 17, 18] with 265 patients reporting frequency of angina attack after treatment favored XST plus conventional medicine. No homogeneity in the results is showed (*P* < 0.00001, *I* ^2^ = 98%). Thus, we did not adopt a meta-analysis. Xiong et al. [13] indicated that frequency of angina attack which decreased from 3.1 ± 1.1 times/day to 0.4 ± 0.5 times/day after treatment favored XST plus conventional medicine. The effectiveness was nearly equal to Kong and Zhang [17] which decreased from 6.72 ± 2.24 times/day to 0.75 ± 0.79 times/day. Another two trials [15, 18] also indicated after treatment favored XST plus conventional medicine: frequency of angina attack decreased from 7.82 ± 1.24 times/day to 2.86 ± 0.72 times/day and from 13.31 ± 0.79 times/day to 4.27 ± 0.87 times/day, respectively.

*(iv) Duration of Angina Attack.* Compared with conventional medicine, three individual trials [13, 14, 18] with 235 patients reporting duration of angina attack after treatment favored XST plus conventional medicine. No homogeneity in the results is showed (*P* < 0.00001, *I* ^2^ = 100%). Thus, we did not adopt a meta-analysis. Xiong et al. [13] indicated that duration of angina attack which decreased from 1.6 ± 0.5 min to 0.11 ± 0.08 min after treatment favored XST plus conventional medicine. Another two trials [14, 18] also indicated after treatment favored XST plus conventional medicine: duration of angina attack decreased from 7.82 ± 1.24 min to 2.86 ± 0.72 min and from 10.23 ± 1.22 min to 4.56 ± 1.08 min respectively.

### 3.4. Publication Bias

Due to insufficient number of trials, we failed to perform funnel plot to detect publication bias.

### 3.5. Adverse Effect

Four of the included trials (66.7%) [14–16, 18] reported adverse effects relating to the treatment by XST combined with conventional drugs. The adverse effects only included rash 0.27% (1/363). No severe adverse events were reported.

## 4. Discussion

In this review, we found that compared with conventional drugs, XST demonstrated a potential beneficial effect for UA on relieving symptoms of angina, improving ECG, and decreasing frequency and duration of angina attack. A total of 6 randomized trials including 716 participants were included. All the RCTs claimed that the positive effect of XST combined with conventional drugs was better than conventional drugs alone. However, we still could not make firm conclusions due to the various durations of treatment and diverse reporting methods in included trials. In addition, the methodological quality of all trials was limited.

The following problems in reporting contribute to the limited methodological quality of most included trials: (1) methods of random sequence generation and allocation concealment: only one trial [13] reported that the random sequence was generated by random number table; most trails were not reported; (2) blinding: blinding of participants and personnel and blinding of outcome assessment were unclear; (3) withdrawal/dropout: one trial [15] reported dropout or withdrawal, but none of the trials had a pretrial estimation of sample size; the detailed reasons of withdrawal/dropout were not reported; (4) information on registration was unclear. We highly recommend further RCTs that should be reported according to the CONSORT Statement [27].

Our systematic review included two major criterions: “ACCF/AHA Guideline for the Diagnosis and Management of Patients with Unstable Ischemic Heart Disease (ACCF/AHA)” and “the International Society and Federation of Cardiology/World Health Organization (ISFC/WHO),” to diagnose UA patients. According to the severity and treatment of diseases, reduction of angina symptoms, ECG improvement, frequency of angina attack, duration of angina attack were used as scales to assess the effectiveness of XST for UA. Four trials [15–18] used reduction of angina symptoms and ECG improvement as outcome measurements. Four [13, 14, 17, 18] used frequency of angina attack and three [13, 14, 18] used duration of angina attack as outcome measurements. The main findings of this systematic review were that XST combined with conventional drugs demonstrated potential effect on relieving symptoms of angina (RR: 1.14 [1.07, 1.22]; *P* = 0.0001) and improving ECG (RR: 1.26 [1.12, 1.42]; *P* = 0.0001) compared to conventional drugs alone. There were only a few trials that report the original data about the frequency and duration of angina; however a substantial level of heterogeneity of these data was indicated and meta-analysis was not adopt.

None of the included trials reported severe adverse events possibly related to XST, and the adverse effects only included rash 0.27% (1/363). We cannot draw firm conclusions about the safety of XST since four of six trials did report information on safety. The sample size was limited, and none of trials had a pretrial estimation of sample size, which indicated the lack of statistical power to ensure appropriate estimation of the therapeutic effect. The duration of treatment in most trials was 4 weeks or 8 weeks, so the potential beneficial or harmful effect of XST for treatment of UA might only result from symptomatic changes and short treatment duration. The included trials used the same doses of XST (two capsules twice a day). Since most participants with UA disease require lifelong treatment, the long-term safety of the treatment is still an important concern. The further studies should pay attention to the monitoring and reporting of adverse events and long-term safety by designing a longer duration of treatment and a long-term followup. Traditional Chinese medicine (TCM) is a holistic system of medicine and has unique theories of the diagnosis and treatment [28–30]. Although XST has been widely used in cardiovascular diseases in China, the efficacy and safety of Chinese patent medicine remain an issue and need evidence to prove it according to the theory of TCM.

## 5. Conclusion

XST in combination with conventional drugs had significant effect on reducing angina symptoms, improving ECG, and decreasing frequency and duration of angina attack with few side effects for patients with UA. However, the findings should be interpreted with caution due to the low quality of included trials. More rigorously designed, large-sample, randomized controlled trials are warranted to support its clinical use in the future.

## Figures and Tables

**Table 1:** The characteristics of included RCTs of Xuesaitong soft capsule for UA.

Study ID	Sample (T/C)	Diagnosis standard	Age	Intervention group	Control group	Course (week)	Outcome measures
Xiong et al., 2006 [13]	89/79	1979 ISFC/WHO	37–68	XST + conventional drugs	Conventional drugs	8	FAA, and DAA
Du and Chen, 2009 [14]	56/56	2002 ACCF/AHA	58.8 ± 9.2	XST + conventional drugs	Conventional drugs	4	FAA, DAA, and adverse event
Yu, 2010 [15]	50/50	2002 ACCF/AHA	64.18 ± 12.13	XST + conventional drugs	Conventional drugs	4	RAS, ECG, adverse event, and Followup
Ge and Zhao, 2010 [16]	48/48	1979 ISFC/WHO	53 ± 2	XST + conventional drugs	Conventional drugs	4	RAS, ECG, and adverse event
Kong and Zhang, 2006 [17]	30/30	1979 ISFC/WHO	36–77	XST + conventional drugs	Conventional drugs	4	RAS, ECG, and FAA
Wei, 2010 [18]	90/90	1979 ISFC/WHO	60.4 ± 3.5	XST + conventional drugs	Conventional drugs	4	RAS, ECG, FAA, DAA, and adverse event

T/C: treatment group/control group; XST: Xuesaitong soft capsule; RAS: reduction of angina symptoms; FAA: frequency of angina attack; DAA: duration of angina attack.
